# Pineapple Juice in Medicine

**Published:** 1902-07-19

**Authors:** 


### PINEAPPLE JUICE IN MEDICINE.

A good deal has lately been written about the digestive action of fresh pineapple. It has been pointed out that a freshly-cut slice of pineapple laid on a piece of beef steak will in a comparatively short time cause softening, swelling, and partial digestion of the meat for a considerable depth from the surface. It is also stated that bromoline the active principle of the pineapple has long been used in the preparation of the well-known Masquera's beef jelly. Dr. Wyatt Wingrave[1] says that the reputation of the pine has suffered, among other reasons, from the facts that far too much is eaten at a time, and that the fibrous part is swallowed as well as the juice. To obtain its full digestive value one quadrant of a slice half an inch thick is ample for one meal. It should be well masticated and the fibrous portion should be rejected. It must not be cooked, and should be just ripe. The preserved form has practically no digestive power. Apart from its use as a digestive, the juice has a strong solvent action upon plastic exudation, such as diphtheria membrane. When applied to such a membrane on a swab, or as a spray, its time of contact is not enough to cause solution, but it is of material service in softening the sticky and stringy exudation so as to admit of its easy detachment. It also softens horny epidermis in the same way as, although more slowly than, salicylic acid. If a thin slice of pineapple be kept in close contact with a corn for eight hours it will be so softened as to admit of ready removal. Again, it softens the horny papillæ in keratosis of the tonsil, and quickly relieves the prickly sensation complained of in that condition.

Evidently the pineapple is possessed of useful as well as of agreeable properties.

[1] Lancet, June 28.

